# Quality-of-Life Evaluation of Patients Undergoing Lumbar Discectomy Using Short Form 36

**DOI:** 10.5812/kowsar.22287523.1998

**Published:** 2011-09-26

**Authors:** Gholamreza Farzanegan, Mohsen Alghasi, Saeid Safari

**Affiliations:** 1 Department of Neurosurgery, Baqiyatallah University of Medical Sciences, Tehran, Iran; 2 Department of Neurosurgery, Tehran University of Medical Sciences, Tehran, Iran; 3 Department of Anesthesiology, Tehran University of Medical Sciences, Tehran, Iran

**Keywords:** Quality of life, Discectomy, Low back pain, Mental health, Laminectomy

## Abstract

**Background:**

Back pain is one of the most common health problems for which physicians are consulted, and it can considerably decrease the quality of life of patients during a great part of their lives.

**Objectives:**

Our study was designed for assessing the improvement in the quality of life of patients undergoing lumbar discectomy for chronic low back pain.

**Patients and Methods:**

We included 148 patients with chronic low back pain in the analytic observational study. Using the 36-Item Short-Form Health Survey (SF-36), we evaluated the quality of life before and 6 and 12 months after lumbar discectomy.

**Results:**

Physical and mental health scores of patients significantly improved after 6 and 12 months of lumbar discectomy. The mean improvement in physical health scores was significantly higher in female patients than in male patients. However, the improvement in mental health scores was not significantly difference between the 2 sexes and the educational and body mass index (BMI) groups.

**Conclusions:**

Lumbar discectomy improves both the physical and mental health subscale of the quality of life in patients with chronic disc herniation.

## 1. Background

Low back pain caused by acute disc herniation is a common disorder among patients in the age group of 20–40 years (1). Back pain is one of the most common health problems for which physicians are consulted, and it can considerably decrease the quality of life of patients during a great part of their lives (1, 2). Patients’ perception of how their illness affects their daily lives can differ from that of their practitioners. Research into the health-related quality of life (HRQOL) has been conducted for only 25 years, but the last decade has witnessed a substantial rise in the level of interest in this field. As a result, HRQOL has become an important and standard outcome for use in health care interventions. It can also be applied as a basis for assessing the health status of populations and to evaluate the burden of a disease by comparing data from clinical groups and the general population (3). It is imperative that clinicians understand how patients experience chronic and often incurable conditions such as chronic low back pain in which the goals of treatment are to optimize HRQOL (5). The 36-Item Short-Form Health Survey (SF-36) was developed in the US as a part of the medical outcomes study (6) and is currently available for the assessment of HRQOL in several countries. This questionnaire has also been validated in Iran (7).

## 2. Objectives

Nowadays, researchers in Iran are focusing on the psychological aspects of therapeutic methods. To this end, we designed our study for assessing the improvement in quality of life of patients who underwent lumbar discectomy for chronic low back pain.

## 3. Patients and Methods

This study was an analytic observational study that was performed by using the cross-sectional method. It was approved by the Baqiyatallah Medical University Ethical Board and was fully supported and funded by Baqiyatallah Medical University.

### 3. 1. Study Samples and Variables

Study samples included 148 patients with chronic low back pain (reported 3 months after disease onset). These patients had been referred to the neurosurgery ward of Baqiyatallah Hospital from January 2009 to May 2010. In this study, we considered variables such as age, sex, educational level, job, height, and weight. Moreover, we collected data on history of abortion, leg pain, back pain, smoking, trauma, pregnancy (including number of pregnancies), driving, sitting for long periods, and lifting heavy objects. Using this data, we evaluated the quality of life of patients before and 6 and 12 months after lumbar discectomy.

### 3. 2. Outcome Measures

We considered the quality of life after laminectomy as the outcome measure in our study. This outcome, i. e. , the patients’ HRQOL, was assessed by using the SF-36 questionnaire, which consists of 36 questions. Not only does this tool yield a total score but it also produces the 2 subscales of physical health (0–100) and mental health (0–100) A higher score suggests a better HRQOL. SF-36 has been previously validated in Iran (7) and has been previously used to compare HRQOL in different chronic conditions (8, 9). Written informed consent was obtained from all patients. All variables were recorded by using the questionnaire.

### 3. 3. Statistics

All data were entered into the computer via the Statistical Package for Social Science (SPSS) software version 14. 0 (SPSS Inc, Chicago, Ill). The aim of our study was to evaluate the quality of life in patients with chronic low back pain. Paired sample t-test was used for evaluating the quality of life before and after lumbar discectomy. One sample t-test was used for comparing the improvement in mental and physical health scores (difference between mental and physical health scores before and after the lumbar discectomy) and the lowest improvement in mental and physical health scores (no change over time in mental and physical health scores) before and after lumbar discectomy. The mean improvement in mental and physical health scores was compared by using an independent sample t-test before and 6 and 12 months after lumbar discectomy on the basis of the patients’ sex, educational level (under diploma or higher), and body mass index (BMI) (under 25 and above that). Two-tailed significance level of 0. 05 was used to detect differences between variables.

## 4. Results

Generally, researchers have focused on the psychological aspect of therapeutic methods and have evaluated them using some variables such as the quality of life. We designed our study for assessing the improvement in quality of life of patients who underwent lumbar discectomy for chronic low back pain. 70 men (46. 6%) and 78 (53. 4%) women were included in our study. Of these patients, 56 (38. 1%) had military jobs, and 77 (53. 4%) had a diploma or higher educational level. Mean age of the patients was 44. 33 ± 11. 53 years. Mean height and weight were 165. 52 ± 11. 17 cm and 75. 21 ± 11. 47 kg, respectively. Mean BMI was 27. 62 ± 4. 39. The mean overall duration of pain between diagnosis and surgery was 34. 61 ± 54. 60 days, and mean duration of pain before diagnosis was 57. 07 ± 68. 50 days (Table 1). 

An evaluation of the history of patients showed that 106 patients (72. 1%) had a history of lifting heavy objects, 101 (68. 7%) of sitting for long periods, 59 (40. 1%) of driving, 20 (13. 6%) of smoking, 75 (51%) of pregnancy, and 22 (15%) of abortion (Table 2). 

**Table 1.** Demographic Variables of Patients with Chronic Low Back Pain

Demographic Variables	
Qualitative variables, No. (%)	
Women	78 (46. 6)
Diploma or higher education	77 (53. 4)
Having a military job	56 (38. 1)
Quantitative variables, (Mean ± SD)	
Age	44. 33 ± 11. 53
BMI ^a^	27. 62 ± 4. 39

^a^ Abbreviation: BMI, Body mass index

**Table 2.** History of Some Activities before Laminectomy Surgery

Activities	No. (%)
Heavy lifting	106 (72. 1)
Sitting for long periods	101 (68. 7)
Driving	59 (40. 1)
Smoking	20 (13. 6)
Pregnancy	75 (51)
Abortion	22 (15)

### 4. 1. Physical Health Score Before and After Lumbar Discectomy

**Figure 1.**
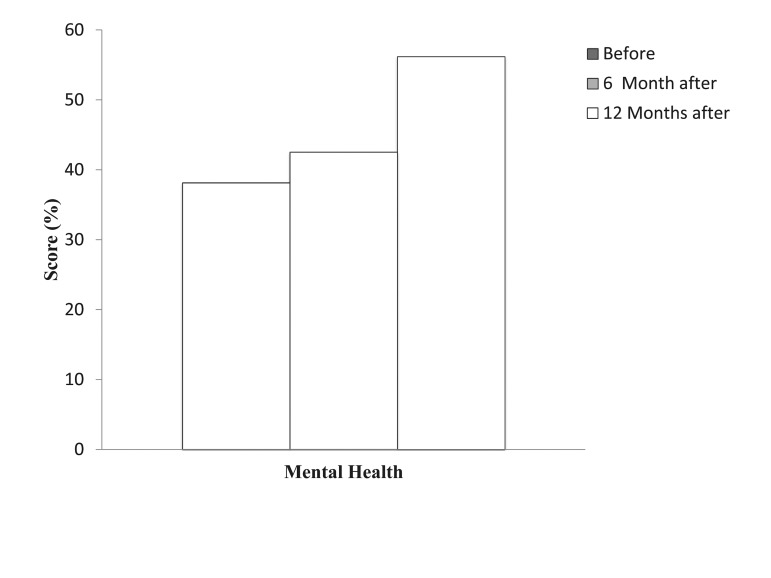
Comparison of Physical Health Score Before and 6 and 12 Months After Lumbar Discectomy

**Figure 2.**
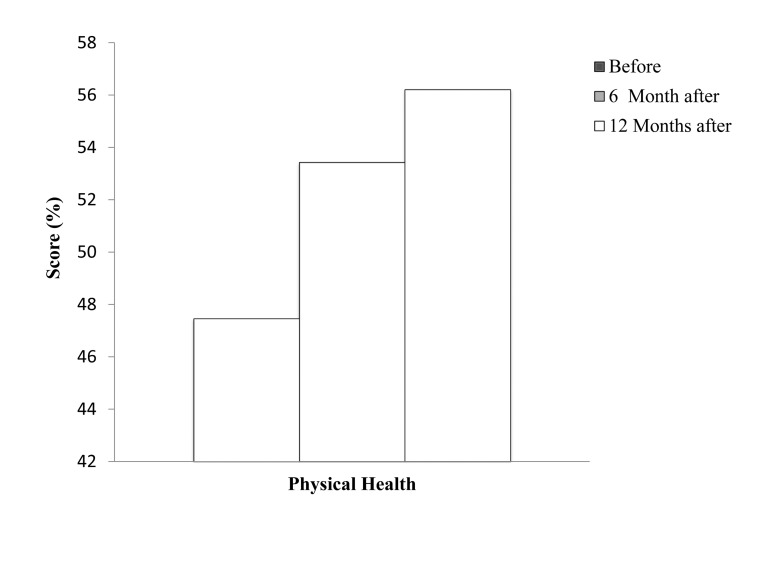
Comparison of Mental Health Score Before and 6 and 12 Months After Lumbar Discectomy

The evaluation of the physical health scores of patients with chronic low back pain showed that the physical health score of patients significantly improved from 47. 46 ± 5. 13 before lumbar discectomy to 53. 43 ± 5. 11 after 6 months (P = 0. 000) and to 56. 21 ± 5. 48 after 12 months (P = 0. 000) of lumbar discectomy (Figure 1, 2).

### 4. 2. Mental Health Score Before and After Lumbar Discectomy

The evaluation of the mental health scores of patients with chronic low back pain showed that the mental health score of patients significantly improved from 38. 16 ± 5. 45 before lumbar discectomy to 42. 54 ± 4. 10 after 6 months (P = 0. 000) and to 43. 48 ± 4. 40 after 12 months (P = 0. 000) of lumbar discectomy.

### 4. 3. Comparison of Improvement in the Physical and Mental Health Scores of Patients Undergoing Lumbar Discectomy and Their Lowest Score

Comparative analysis showed that the mean improvement in the physical health score 6 months (5. 97 ± 7. 65; P = 0. 000) and 12 months (8. 76 ± 7. 98; P = 0. 000) after lumbar discectomy was significantly higher than the lowest improvement in physical health score. Moreover, the mean improvement in the mental health score 6 months (4. 38 ± 7. 10; P = 0. 000) and 12 months (5. 32 ± 7. 13; P = 0. 000) after lumbar discectomy was significantly higher than the lowest improvement in mental health score.

### 4. 4. Comparison of Physical and Mental Health Improvement in Patients with Chronic Low Back Pain According to Sex

The mean improvement in the physical health score of female patients (10. 79 ± 7. 14) was significantly higher than that in male patients (6. 51 ± 8. 30; P = 0. 000); the improvement in mental health scores of female patients (6. 29 ± 7. 40) and male patients (4. 26 ± 6. 73) showed no significant difference (P = 0. 080).

### 4. 5. Comparison of Physical and Mental Health Improvement in Patients with Chronic Low Back Pain According to Educational Level

The mean improvement in physical health score showed no significant difference in the 2 educational groups (under diploma, 9. 40 ± 7. 23 and higher than diploma, 8. 17 ± 8. 61; P = 0. 350). The mean improvement in mental health score in the under diploma group (5. 66 ± 7. 45) and higher than diploma group (5. 01 ± 6. 87) showed no significant difference (P = 0. 590).

### 4. 6. Comparison of Improvement in the Physical and Mental Health Scores in Patients with Chronic Low Back Pain According to BMI

Mean of physical health score improvement hadn’t significant difference between two BMI groups (under 25 and above that). (8. 38 ± 7. 93; 8. 93 ± 8. 04; P = 0. 680). Moreover, the mean improvement in mental health scores of the 2 BMI groups under 25 (6. 25 ± 7. 27) and above 25 (4. 92 ± 7. 07) were not significantly different (P = 0. 310).

## 5. Discussion

We included 148 patients in our study. The physical and mental health scores of patients 6 and 12 months after lumbar discectomy were significantly higher than those before lumbar discectomy. Mental health improvement in men and women was not significantly different. The mean improvement in physical and mental health scores showed no significant differences between the 2 educational and BMI groups. Lumbar disc herniation is the most common disease caused by spinal degenerative processes and accounts for 30–80% of the low back pain cases (10). The interest in the use of HRQOL measures for assessing the outcomes of spinal surgery has been increasing because it might allow comparisons across studies by using standard questionnaires (11-14). One of the most frequently used questionnaires for the evaluation of HRQOL in patients with spinal pathological conditions is the SF-36 (15). The SF-36 is advantageous in that it achieves the best balance between length, reliability, validity, responsiveness, and experience, even in large populations of patients who complain of low back pain (12, 16).

For the complete assessment of benefits of a surgical intervention, it is essential to provide evidence of the influence over the patient in terms of the health status and HRQOL (17, 18). These terms refer to symptoms of illnesses such as pain and fatigue and to broader aspects of the individual’s physical, emotional, and social well-being (19). Unlike conventional medical indicators, these indicators of physical, emotional, and social well-being and treatment need to be assessed and reported by the patient (20, 21). Therefore, the application of patient-assessed measures of health outcome has become increasingly important for health care evaluation (17). Quality of life is a critical outcome measure, but the best way to measure it is unclear (22-24). On the basis of these studies, Lang et al. and Epstein and Hood found that after complex neurosurgery and lateral lumbar disc surgery, surgeon-assessed outcomes correlated poorly with the SF-36 scores and that surgeons underestimated the impact of neurosurgery on patients’ quality of life (4, 25).

Our study had some limitations. First, we assessed the quality of life scores by using the SF-36 questionnaires; other methods may show different results. Second, the study was restricted to the Iranian population. Third, this study was conducted at a single center, and hence, the external generalizability of our findings to other countries or centers is uncertain. Lumbar discectomy improves both the physical and mental health subscale of the quality of life in patients with chronic disc herniation.
